# Commercial activities and subsistence utilization of mangrove forests around the Wouri estuary and the Douala-Edea reserve (Cameroon)

**DOI:** 10.1186/1746-4269-5-35

**Published:** 2009-11-17

**Authors:** Adolphe Nfotabong Atheull, Ndongo Din, Simon N Longonje, Nico Koedam, Farid Dahdouh-Guebas

**Affiliations:** 1 Laboratoire de Complexité et Dynamique des Systèmes Tropicaux, Département de Biologie des Organismes, Faculté des Sciences, Université Libre de Bruxelles - ULB, CP 169, Avenue F.D. Roosevelt 50, B-1050 Bruxelles, Belgium; 2 The University of Douala, Faculty of Science, Department of Botany, PO Box 8948 Douala, Cameroon; 3 Biocomplexity Research Focus c/o Laboratory of Plant Biology and Nature Management, Faculteit Wetenschappen, Vrije Universiteit Brussel - VUB, Pleinlaan 2, B-1050 Brussels, Belgium

## Abstract

**Background:**

Worldwide there is growing research interest in the ethnobiology of mangrove forests. Notwithstanding that, little information has been published about ethnobiology of mangrove forests in Cameroon. The aims of this study were a) to analyze the harvesting methods and the local selling of mangrove wood products by loggers in the vicinity of Wouri estuary and b) to investigate the patterns of subsistence uses of mangrove wood products around the Douala-Edea reserve.

**Methods:**

Semi-structured interviews were conducted with 120 active mangrove loggers in 23 Douala wood markets and 103 households located in three villages (Mbiako, Yoyo I and Yoyo II) close to Douala-Edea reserve. In each of the three densely populated villages, every second household was chosen for sampling while in all markets, mangrove loggers were chosen randomly. In addition, log diameters were measured in each market using a wooden foldable tape measure. A *post hoc *analysis (Newman-Keuls test) was performed in order to detect the common wood class diameter sold in the Douala wood markets.

**Results:**

The analysis of the loggers' survey data has shown that large logs of *Rhizophora *with diameter greater than 40 cm were common in the Douala wood markets and were more closely associated with loggers who used chainsaws. In addition to the general mangroves wood products selling, the analysis on a subsistence level (households' survey) suggests the local population's dependence on mangroves, with multiple uses of *Rhizophora racemosa *Meyer, *R. harrisonii *Leechman, *Avicennia germinans *L. Stearn., *Laguncularia racemosa *Gaertn. f. and *Conocarpus erectus *L. timbers for furniture, fences, smoking fish, and fuelwood. Finally, *Nypa fruticans *(Thunb.) Wurmb. leaves were used as thatching material for house walls and roofs.

**Conclusion:**

Our findings revealed that big logs of *Rhizophora *were commonly sold by the loggers. A majority of loggers (60%) reported that mangrove marketed wood constitute a principal source of income. Most of the villagers (85.83%) often depend on mangroves for subsistence needs and for them there is no substitute for mangrove wood. Therefore, more efforts should be undertaken at the national level to implement conservation, management and sustainable use of these coastal forests.

## Background

Throughout the world, mangroves are among the most productive ecosystems because of their exceptional flora and fauna diversities [1-6]. These forested wetlands are socio-economically and ecologically important for local communities who use them amongst others as a source of wood and non-wood forest products or as a living space [7-13]. They fulfil a habitat function for a variety of commercial fish and shellfish species that breed, spawn, hatch or develop in the mangrove. They also act as living dykes by protecting coastal communities against the effects of wind, waves and water currents. Despite the aforementioned roles of these coastal ecosystems, their annual loss rate is still high (about 3.6 million hectares has been lost since 1980) [4,14,15]. To date coastal economic development still remains the main cause of global mangrove decline worldwide [15-19]. The threats are significant since human migration into coastal zones is continuously increasing and the majority of people living in or near mangrove areas are poor [13].

In Cameroon, very little attention is paid to mangrove forest management, despite the fact that this country has the third largest mangrove area in West-Africa after Nigeria and Senegal [14,20]. In these countries, human disturbances caused severe damage to mangrove ecosystems and threaten them [20]. Many disturbances are obvious [7,21,22] and can be easily observed in some mature stands of Cameroon mangrove forests were variety of anthropogenic activities have largely modified the microtopography. This in turn may affect mangrove dispersion and establishment [23], early development [24], growth [25] or vegetation dynamics [26]. But some disturbances, such as cryptic ecological degradation [27], are particularly well hidden to show their terrible consequences only in case of natural hazards [28].

The high productivity [29-31] and proximity of mangroves with large (sub)tropic urban centres has favoured human establishment in or near these lush forests, examples include cities such as Manila (Philippines), Colombo (Sri Lanka), Mombasa (Kenya), Banjul (The Gambia) and Douala (Cameroon). In the latter case, like elsewhere, patterns of harvest reflect the spatial distribution and relative accessibility of mangroves, which varies depending on local geomorphology and hydrology, socio-economic conditions and past human disturbance [32,33]. Although the dense prop-roots tend to make access to and clearing of mangrove forests difficult [11,13], tree stems are still harvested and fisheries products collected there by local communities.

The overexploitation (cutting of several small and big trees in different stands) and clear-felling (clear cut of all trees in a larger area) of mangrove plants is rarely a full-time occupation for local communities [14,34-41] but the use of chain saws in the logging operations is clearly the main factor that impacts mangrove cover in the Cameroon estuary [11]. Informal activities such as fishing, hunting [13], and sand and gravel extractions also contribute to the degradation of mangroves in this wetland ecosystem.

Although research suggests that harvesters are often flexible in their preferences [3,33], high-scale mangrove logging (extensive cutting of big logs in different areas) encourage by the growing demand in wood products at Douala markets have led to heavily impacted stands within the mangrove forests. However, despite the complex relationship between coastal communities and mangrove, only few ethnobotanical surveys in mangroves of Cameroon estuary have been conducted [11].

The present study was undertaken in the vicinity of Douala (Wouri estuary) and in three villages adjacent to the Douala-Edea reserve (Mbiako, Yoyo I and Yoyo II). The major objectives were a) to analyze the harvesting methods and the local selling of mangrove wood products by loggers in the vicinity of Wouri estuary and b) to provide understanding in the patterns of mangrove wood product utilization by local people (villagers) inhabiting the aforementioned villages. Attempts were also made to estimate the importance of mangrove for the local communities and to assess their perception on the evolution of mangrove forests during their life time.

## Methods

### Study area

**Figure 1:**
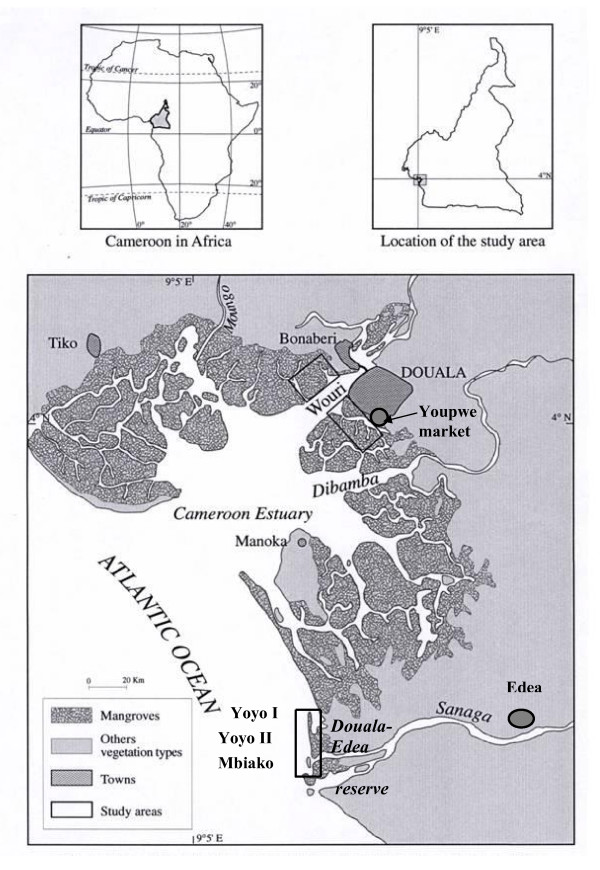
**Mangrove of the Cameroon Estuary [modified from Din et al. **[42]**].**

This study was conducted at Cameroon estuary, located in the Gulf of Guinea (3° 40'- 4° 11' N and 9° 16' - 9° 52' E). It is estimated that mangrove cover 1100 km^2 ^in this estuary (Figure 1) [14]. Throughout the year, rainfall in the region is abundant (about 3988 mm) and the average annual temperature is high (26.7°C). The climate is of the particular equatorial type, so-called "*Cameroonian*" [42]. Strong tidal influences on rivers (Wouri, Dibamba and Sanaga) and freshwater influxes enable mangroves to grow as far as 100 kilometres inland (Figure 1) [20]. Mangroves around Douala, Mbiako, Yoyo I and Yoyo II often appear as dense and big trees with a canopy reaching 30-40 metres in height. Cameroon's estuary mangrove forests are continuously under higher human pressure because of the increasing demographic patterns in the adjacent urban areas like Douala where the total number of active loggers has been estimated to be 350 [11]. There were no official statistics available about the village populations of our study area. Nevertheless, the village chiefs estimated this number to be about 1,845. Although we acknowledge that this number is relatively low, the human disturbance is continually greater in the mangrove forests close to Mbiako, Yoyo I and Yoyo II villages. The latter villages were selected because of their vicinity and strong interaction between their relative dense communities and the mangrove forest. Whereas Yoyo II and Mbiako sites are characterized by a medium size, the Yoyo I village is much stretched. Consequently, this has led to a variation of sample size in different villages (22 in Mbiako, 54 in Yoyo I and 27 in Yoyo II).

### Loggers' survey

The first field trip was carried out in November 1999 and March 2000 in 23 Douala wood markets. The logger survey sheets comprised eight sections (Table 1). Questionnaires were of semi-structured type [9] with short proposed answers or free open answers. In the surveyed wood markets, mangrove loggers were chosen randomly. In case where informants chosen were the resellers (in a few case, some loggers delivered big logs to various resellers who are often paid by the loggers after the sale), we often recorded information from the logger who has engaged the reseller. This procedure was pursued since loggers were often the vendors and therefore mostly indicated to provide both responses regarding harvesting and wood selling (the price of marketed wood is usually defined by the loggers). A total of 120 active mangrove loggers (comprise of only men) were interviewed. Based on the total number of loggers existing in the population [11], we assumed that 34.28% of active loggers were surveyed. In addition to survey, we asked the loggers for permission to measure the diameter of logs by using a wooden foldable tape measure. By doing so, we also estimated the length of the logs.

**Table 1:** Broad sections of topics constituted of clusters of questions dealing with these topics for mangrove loggers in 23 urban wood markets in Douala

A. Survey with loggers	
**A1**. The social situation (the agreement of the surveyed logger is mandatory) allows the logger to be identified, details his/her past, present and future activities, and the sources of energy he/she is using. This was asked at the end of each interview.	**A2**. The localization of the exploitation site which concerns at the same time the fuel wood logging points in the field, and the markets in which the produce is sold.

**A3**. The resources used provide information on the tools the mangroves loggers possess and/or use during their activity, including such tools as canoes, engine-powered canoes or boats, chain saws, carpenter saws, machetes, axes, etc.; B3. The general utilisation of mangroves gave a first idea of uses in general. Although no further focus, here we inquired also for non-wood forest product uses such as medicinal or alimentary utilization of the mangrove.	**A4**. The daily production is a set of raw numbers provided by each logger. This is the fundamental part of this survey that should provide answers about deforestation and estimates the economic value of the mangrove wood resources. It specifies the targeted species, provides the number of trees logged in the field, usually the length of the used part of the fallen tree, as an alternative to the knowledge of the tree heights, the number of logs obtained and their diameter, and the transported quantity. The surveyor did not adjust or modify the information obtained from the mangrove loggers. B4. Detailed questions on fuelwood use investigating genera used, their quality, their part and size used. We also asked for reasons of use and for any alternative fuelwood uses. Who and how much was used was also part of the survey.

**A5**. The expenditures of the mangrove logger shed light on the overall necessary resources for the accomplishment of this activity. They concern the equipment that he/she can rent or the labour used, the cost of food eaten in the field, different types of fuels, reseller remuneration, etc.	**A6**. The daily incomes of the mangrove logger are inferred from the answers received on the average quantities of daily sales in the market, while taking into account the eventual price fluctuations of all the markets according to the universal law of demand and supply.

**A7**. The monthly revenues are a statement provided by the mangrove logger, without any relation with the aforementioned revenues and expenditures.	**A8**. The role of the mangrove is restricted to a unique question that concerns the environmental knowledge of the mangrove logger.

### Households' survey

The second field survey was carried out between February and March 2002 in three villages near the Douala-Edea reserve: Mbiako (3°30'N, 9°39'E), Yoyo I (3°38'N, 9°38'E) and Yoyo II (3°40'N, 9°38'E). The questionnaire sheets contained seven sections (Table 2). The survey was done amongst regular inhabitants in the three aforementioned villages by conducting interviews using semi-structured questionnaires with 103 villagers (comprised of men or women representing a household). In each village, households were considered as basic sampling units. In order to avoid repetition from members of the same household, only one person per household was surveyed [9]. Over the three densely populated villages, every second household was chosen for sampling. However, information was not recorded in the all households that were chosen because of uncomfortable reception or hostility. In this case and when information gathered in the previous household was incoherent, we sampled in the adjacent one.

**Table 2:** Second sections of topics constituted of clusters of questions dealing with these topics for mangrove villagers in three villages adjacent to the Douala-Edea reserve

B. Survey with villagers	
**B1**. Socio-demographic and economic attributes of the villager (incl. age, religion, marital status, household composition, years of life in village, profession, family income source, and assets of the family). This was asked at the end of each interview.	**B2**. An objective assessment of the interviewee's knowledge on mangroves (in the text referred to as 'mangrove knowledge') was made by asking the person to identify which mangroves were around using plants parts freshly collected from the field and scoring the correctness as described by Dahdouh-Guebas et al. [9].

**B3**. The general utilisation of mangroves gave a first idea of uses in general. Although no further focus, here we inquired also for non-wood forest product uses such as medicinal or alimentary utilization of the mangrove.	**B4**. Detailed questions on fuelwood use, investigating genera used, their quality, their part and size used. We also asked for reasons of use and for any alternative fuelwood uses. Who and how much was used was also part of the survey.

**B5**. The same was repeated for construction and service wood.	**B6**. The same was repeated for fine timber (for furniture, crafts and arts).

**B7**. Personal assessment of the interviewee's perception on changes in the mangrove forest was gained by asking questions on change in area, in mangrove species composition and on opinion reasons for this. We also asked them about their opinion of the future.	

In each of the households sampled (103), the average number of persons was 4.65 ± 2.16. Based on total population estimates from the Village Chiefs (Table 3), we calculated that the total number of households of Mbiako, Yoyo I and Yoyo II was about 132, 158 and 86, respectively. Therefore 16.66%, 34.17% and 31.39% of households were sampled at Mbiako, Yoyo I and Yoyo II, respectively. We draw the attention to the second section (Table 2, B2) which investigates each respondent's knowledge on mangroves, hereafter referred to as 'mangrove knowledge'.

**Table 3:** Descriptive statistics of the total population structure within the villages surveyed (a), marital status (b) and profession (c) of the respondents.

	Mbiako	Yoyo I	Yoyo II	Total
**Number of questionnaires**	22	54	27	103

**Socio-demographic factors**				

**(a) Population structure (based on total population statistics from the Village Chiefs)**				

Adult Male	294	240	87	621
Adult Female	108	264	120	492
Child	213	234	195	732

**(b) Marital status of respondents**				

Married	11 (50%)	29 (54%)	16 (59%)	56 (54%)
Bachelor	7 (32%)	20 (37%)	8 (30%)	35 (34%)
Widow	4 (18%)	5 (9%)	3 (11%)	12 (12%)

**(c) Profession of respondents**				

Business	8 (5%)	12 (22%)	7 (26%)	27 (26%)
Fishing	16 (73%)	38 (70%)	15 (56%)	64 (62%)
Smoking fish	11 (50%)	41 (76%)	17 (63%)	74 (72%)
Other (*e.g*. teaching, *ad interim *jobs)	1 (5%)	2 (9%)	0 (0%)	3 (3%)

The values in parentheses indicate percentages per village or for all villages together (Total) for a particular demographic factor

Note that they do not always necessarily add up to 100%.

### Analysis of the loggers' survey

Quantitative and qualitative data provided by loggers were encoded and analyzed differently.

#### Quantitative analysis

The loggers' age, ranging from 20 to 69 years old, was split into five equal classes according to a normal distribution. The first and fifth classes refer to youngsters and elderly, respectively, whereas the three others classes were found to comprise (middle-aged) adults (see results). The years of experience in logging activities were also divided in four classes of 10 years interval based on socio-economic criteria. The criterion was the 1990s (10 years before we sampled) economic crisis that has led to the increasing human migration toward cities such as Douala (Cameroon) which is adjacent to mangrove forests. One-way ANOVA was performed to determine the difference in monthly income between age classes and wood diameter classes. This statistical analysis was most preferable as we were dealing with comparisons of more than two quantitative data. A *post hoc *analysis was afterward done with Newman-Keuls test to detect the common wood class diameter sold in the markets. Differences between wood quantities cut down and the one transported to the market were also analysed using the Student t-test.

#### Qualitative analysis

The exploitation equipment is a determinant for the size, quality and number of the trees that can be cut down by loggers. Different materials used for mangrove deforestation have been identified during the survey and their classification has been made in relation to their acquisition mode (owned, shared or rented). Therefore, impact of this equipment on the mangrove degradation and household income can be appreciated. Information concerning the diverse form of wood sold, the different people involved and their years of experience in this commercial activity were provided by respondents encountered in each market.

### Analysis of the villagers' survey

Because of the different nature of the purpose in the surveys, the villagers' survey was not analysed in the same way as the loggers' survey. The villagers' survey was primarily descriptive, but some relevant answers were confronted with the mangrove knowledge of the respondents.

## Results

During fieldwork, loggers and villagers were relatively open to provide information about resource extraction patterns and changes that have occurred within mangroves. Nevertheless, the accuracy of responses was considered significant when they were able to identify mangrove species.

### Loggers' survey

#### Mangrove wood sale

The age of mangrove sellers ranges from 20 to 67 years with average of 45 ± 8 years. Among them (120 loggers), 60% considered marketed woods as their principal source of income. Likewise, about 19% of these loggers were currently involved in informal job practices such as railway workers, mechanics, motorcycle drivers, bricklayers, security guard agents, charcoal makers and soap manufacturers. Their entire income was used to ease the household expenditures. Thirty-eight percent (38%) of loggers interviewed, mostly young people awaiting a formal job, sold mangrove wood temporarily. Only 2% of mangrove loggers were retired. They often used revenues provided by mangrove products in complement to their retirement allowance.

**Figure 2:**
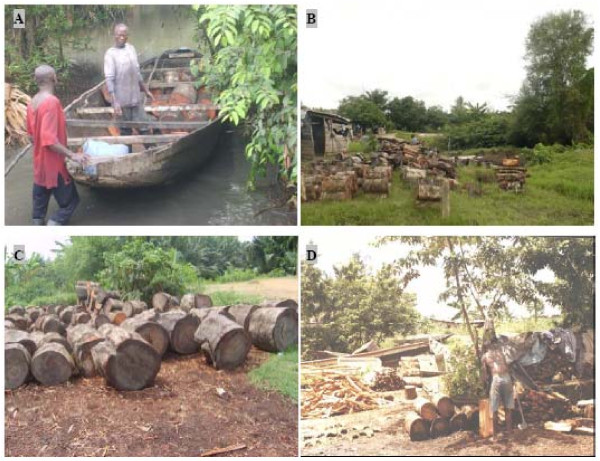
**(A) Unloading of mangrove wood near the Douala wood market (Youpwe)**. **(B) Mid-size logs and small timbers commercialized at the Douala wood markets. (C) Big marketed logs at the Douala wood markets (D)**. (Photograph by ANA). **Transformation of big logs into heaps (this wood is sold at local markets *e.g*. near the Wouri bridge)**. (Photograph by ND).

The loggers reported that mangrove logs were often taken in stands that were closer to the city and easiest to access by creek. Large and small *Rhizophora racemosa *Meyer and *R*. *harrisonii *Leechman timbers, cut down with chain saws or machetes and carpenter saws respectively, were commonly transported towards the local wood markets using traditional boats (Figure 2A). At the markets, only logs with diameter < 40 cm were traded in form of heaps. A heap of 3 logs (equal to a cubic meter) with a length of 60 cm and a diameter comprised between 20-40 cm cost 2.85 € (in the 1999s, 1 € = 655.95 FCFA or Cameroon Franc) (Figure 2B, on the left) while 10-15 logs assembled (equal to a cubic meter) with a length comprise between 60-80 cm and a diameter < 20 cm cost about 2.30 € (Figure 2B, on the right). On the other hand, a large log with a length of 35 cm and a diameter greater than 40 cm (Figure 2C) cost about 4.60- 7.62 € (this price was equal to 3000 - 5000 FCFA). Big logs bought were often transported towards the centre of Douala for selling or heating of the henhouse. In some cases, however, logs were split into pieces and afterward assembled in form of small heap for sale (Figure 2D). A heap of four mangrove wood pieces cost on average 0.20 ± 0.019 €. It was used by the buyer for cooking, heating or making charcoal.

**Figure 3:**
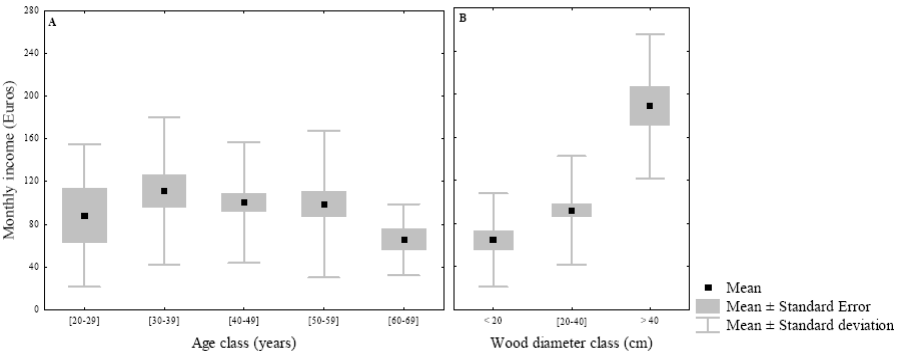
**(A) Repartition of loggers' monthly income between age classes**. Elementary statistical analysis also showed that average monthly income of the third class was not significantly different from the one of the first class (t = 0.48; df = 51; p = 0.62), the second class (t = -1.02; df = 66; p = 0.30), the fourth class (t = -1.44; df = 45; p = 0.15) and the fifth class (t = -1.44; df = 45; p = 0.15). (B) Relation between revenues and wood diameter sold at all markets visited.

In the vicinity of Douala, the trade of mangrove wood products is common. The loggers have declared average monthly revenues in the order of 98 ± 61.41 €. There were not significant differences of average monthly income between logger age classes (f = 1.14; df = 4; p = 0.34) (Figure 3A). However the average quantity of wood transported to market by loggers of the fifth age class was significantly lower than that of the others age classes (f = 2.55; df = 4; p = 0.04). Further statistical analysis also revealed that mangrove wood diameter of the third class was significantly more sold at wood market than the one of the first class (t = 7.63; df = 73; p < 0.0001) and the second class (t = -6.71; df = 91; p < 0.0001) (Figure 3B).

#### Diameter of mangrove wood at markets

**Figure 4:**
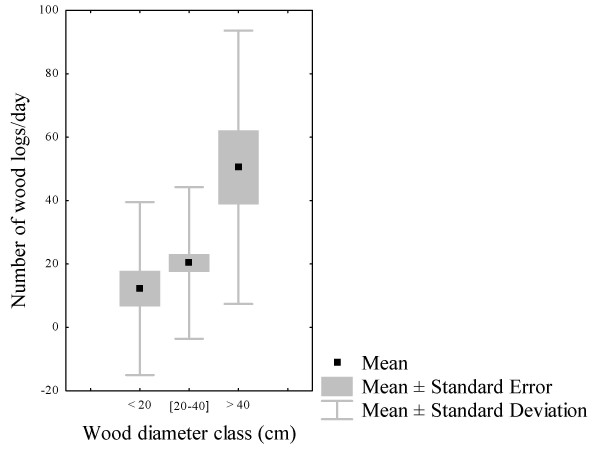
**Box and whisker showing the reparation of the number of wood logs and their diameter**.

**Figure 5:**
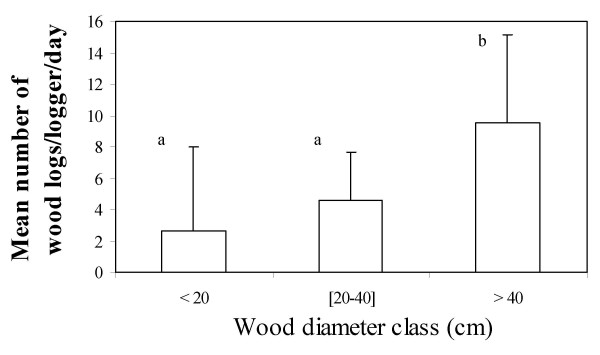
**Histogram of the average number of trunks cut down and their diameter class (the vertical bars indicate the standard deviation whereas the letters differentiate between wood diameter classes most exploited (b) and less exploited (a)**.

During cutting of logs, loggers often used chain saws to cut down large trunks whereas carpenter saws and machetes were used when harvesting small and tall trees (Table 4). The need of loggers to increase the household income thrusts them to claim or rent this kind of equipment. The average diameter and length of the logs were 41.36 ± 11.92 cm and 43.28 ± 12.26 cm, respectively. The survey revealed that there was a highly significant difference between diameter classes of wood exploited (f = 12; df = 2; p = 0.000018) (Figure 4). A *post-hoc *analysis (Newman-Keuls test) showed that trees with an average class diameter greater than 40 cm were preferred over the two others classes which were all harvested in the same way (Figure 5). Although big logs were uncommon outside the regular wood markets, the largest diameters of wood products were found with the loggers who use chain saws.

**Table 4:** Acquisition mode of exploitation materials used for wood cutting in Cameroon estuary and their impact on mangrove degradation and household income

Equipment	Users number	Acquisition mode		Impact on	
			
		Owners	Rented	Mangrove degradation	Household income
**Boat**	120	94 (78.33%)	26 (21.6%)	Average	Positive
**Chain saws**	31	18 (58.1%)	13 (41.9%)	Very high	Positive
**Carpenter saws**	103	103 (100%)	0 (0%)	High	Positive
**Machetes**	120	120 (100%)	0 (0%)	Little	Insignificant

Five trees on average (4.75 ± 4.36) were cut down daily by each harvester. *Rhizophora racemosa *was heavily harvested because of its availability, dominance and suitability for making firewood and charcoal. On the other hand, in regard to the diameter of wood products, mangrove logging was often non-selective. Many small timbers of *Rhizophora *spp., often used as building material, were also found in the local markets. The same was true for species such as *Conocarpus erectus *L. and *Avicennia germinans *L. Stearn. Generally, the entire quantity of wood harvested was not transported to the market on a daily basis. Nonetheless, only a small amount of large trunks were left in the field. Therefore, the average quantity of wood logs by loggers was not significantly different from the one transported at the market (t = 1.38; df = 17; p = 0.18).

#### Years of experience in logging activities

**Figure 6:**
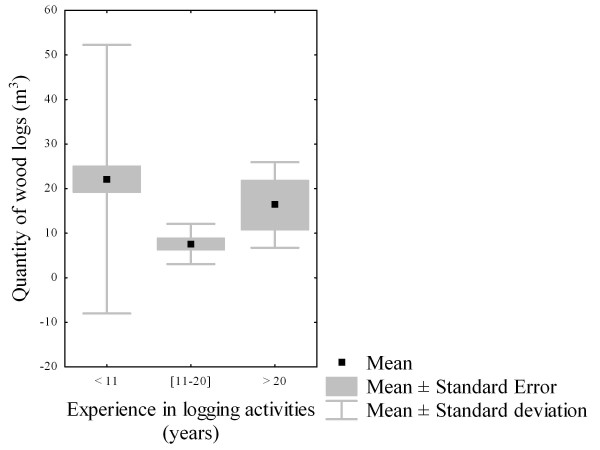
**Distribution of the quantity of wood logs according to experience in logging activities**.

**Figure 7:**
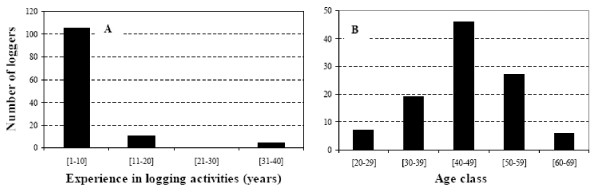
**(A) Repartition of loggers between logging experience classes**. (B) Distribution of loggers with ten years of experience in logging activities and their average income by age groups.

Since the loggers were not involved in logging every day, their years of experience in this activity were considered as relative. We did not find differences in quantity of wood logs between years of experience classes (f = 0.32; df = 2; p = 0.72) (Figure 6). Although local forest users with greater experience (more than 10 years) in logging (Figure 7A) were less represented (15/120), they were more likely able to know where stands with large trunks could be found. Furthermore, their skills during handling operation and boat manipulation allowed them to heavily collect mangrove wood products. However, most of the loggers (105/120) have been harvesting mangrove wood for 10 years (Figure 7A). They were found to be adults and their physical strengths allowed them to widely over-cut mangroves. Amongst them, 43.82% were between 40 and 50 years old (Figure 7B). While carrying out logging activities, they were looking for alternative jobs and were willing to abandon wood harvesting if they were hired elsewhere. On the order hand, 18.09% (19/105) and 25.71% (27/105) of loggers with 10 years of experience belonged to the second (30-39 years) and the fourth (50-59 years) age classes, respectively. Only 6.67% of loggers were youngsters (20-29 years) and 5.71% were in the elderly class (60-69 years).

### Villagers' survey

#### Ethno-botanical knowledge of mangroves

The majority of respondents was married and was mostly involved in fishing, fish smoking or both (Table 3). Understanding the respondents' mangrove knowledge was the initial step to make sure data interpretation was sound. There was a group of respondents who had expert mangrove knowledge about the different species of mangroves and were able to describe them by leaf structure, bark and flowers separately. This category represents 10% of the survey. Another group, the majority (50%), could only distinguish between mangrove species by describing the leaves, and we term them as having a 'good working knowledge'. Yet, another group of respondents could make distinction between mangroves when assisted by samples of the species (26%). The final group had no idea about mangrove species, but they knew what the mangrove forest was (14%). However, they were unable of describing their characteristics.

#### General utilization of mangroves

Five villagers interviewed were also loggers. They were native people and were not involved in marketed woods. About 3 trees were cut weekly by each villager for domestic use. The different mangrove species present had different uses. *Avicennia germinans *was used for furniture, fencing poles, fuelwood for cooking and smoking fish, bed poles, timber poles for *banda *(table-like construction to smoke fish) construction, canoe anchors, paddles and fishing traps (Table 5).

**Table 5:** Subsistence uses of mangrove by local people around three villages (Mbiako, Yoyo I and Yoyo II) established within Cameroon estuary

Taxa name	Part used	Uses
***Rhizophora *spp.**	Young, mature and old stems, branches	Fuelwood (cooking and smoking fish), furniture, fencing poles, fuelwood for cooking and smoking fish, bed poles, timber poles for *banda *construction, canoe anchors, paddles and fishing traps bridges.
***Avicennia germinans***	Young, mature and old stems, branches	Fuelwood (cooking and smoking fish), furniture, fencing poles, fuelwood for cooking and smoking fish, bed poles, timber poles for *banda *construction, canoe anchors, paddles and fishing traps.
***Laguncularia racemosa***	Stems	Firewood (home use), smoking fish, poles for furniture and fences.
***Conocarpus erectus***	Stems	Firewood (home use), smoking fish, poles for furniture and fences.
***Nypa fruticans***	Leaves	Thatching material for house walls and roofs.

**Figure 8:**
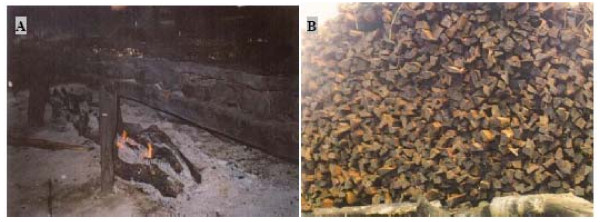
**(A) *Banda*, used to smoke fish on**. (B) Stacks of mangrove wood ready to be used as *banda *and to be burnt for smoking the fish. Mangrove wood is thus used to construct the table-like *banda *but also to burn and smoke the fish itself. (These photographs were taken at Yoyo I by SNL).

**Figure 9:**
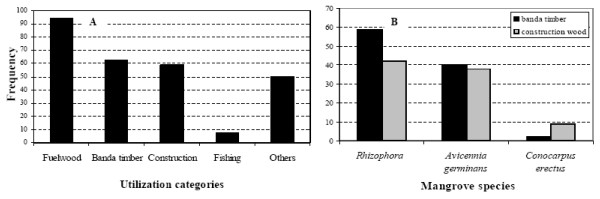
**(A) Utilization frequency of different mangrove use categories around the Douala-Edea reserve**. (B) Mangrove species used in construction, either as *banda *timber (black) or as construction wood (grey). (The genus *Rhizophora *mostly used is represented by *R. racemosa *and *R. harrisonii*).

Young, mature and old stems were used for this, and so were branches. The same parts were used from *Rhizophora racemosa *and *R*. *harrisonii *for the same purposes (Figures 8A, B). In addition, they used *Rhizophora *spp. poles for bridges. Stems of *Laguncularia racemosa *Gaertn. f. and *Conocarpus erectus *were used as fuelwood for home use and for smoking fish, and as poles for furniture and fences. Finally, *Nypa fruticans *(Thunb.) Wurmb. leaves were used as thatching material for house walls and roofs. The most common use of mangroves was for fuelwood (Figure 9A), which was particularly true for *Rhizophora *spp. because of their availability, and their slow, highly calorific and smokeless burning properties. Within the two construction uses, *i.e. banda *timber and construction wood, *Rhizophora *spp. and *Avicennia germinans *have a significantly higher preference than *Conocarpus erectus *(Figure 9B). The majority of the respondents (101 over 103) indicated that there was no substitute for mangrove wood or at least they did not know about it.

#### Perception on changes in the mangrove

When asked whether the mangrove was very, little or not important, a wide range of respondents (87%) with expert and good mangrove knowledge answered that the ecosystem was of prime importance. Nevertheless, fifty six percent of the respondents reported that there was a negative change in the mangrove. Among them, 60% indicated that the decline was serious and 40% reported that it was average. Respondents related the decline to selective harvesting and uncontrolled deforestation. It was reported that every family has the right to harvest mangrove wood at anytime, anywhere and in any quantity. However, this appeared to be just a perception as Cameroon legislation protects mangroves as a particular ecosystem (Frame-law n°96/012 relative to management of environment in Cameroon, august 5^th ^1996). Others maintained that local population growth and the resulting pressure on coastal zone have led to a decrease in the extent of mangrove area. Interestingly, one respondent reported that due to a lack of adequate technology most of the heat produced during fish smoking is wasted.

## Discussion

### Commercial uses

The mangroves of Cameroon are under legal protection but this has not effectively precluded local residents from continuing to harvest them since the protective law is not well enforced. The rapid growth of human populations and the resulting pressure on coastal environments often lead to uncontrolled logging, posing severe threats to the mangroves [15]. Negative change rates in the extent of mangrove surface area of this region have been reportedly aforementioned. As in Indonesia, commercial exploitation of mangrove wood has been important and therefore considered as one of the current threats [15]. Besides extensive felling of mangrove plants in the Wouri estuary, oil and solid pollution also negatively affect the health and quality of the stands. Some examples have been found in the Manoka bay (southern Cameroon) where, even though banned, mangroves have undergone an industrial exploitation at the dawn of last century [43]. The signs reminding of this activity are still visible to date especially were a wood processing plant and the port for export was located [11].

On the other hand, however, exploitation of mangroves in the Wouri estuary was an activity predominantly undertaken by local residents. These coastal ecosystems are traditionally used for the production of firewood, charcoal, boats, fish-traps, timber and poles for houses. The commercial exploitation of mangrove wood products around the Wouri estuary is local, common and well-developed. For instance, sales of mangrove wood were consistently observed at all markets visited. Large mangrove timbers, usually harvested with chain saws, are transported to the market using traditional boats (non-engine-powered canoes). In spite of drowning risk, low swimming ability of loggers and lack of protective material (life vests) most of harvesters interviewed (60%) were not dishearted because marketed wood appears to be very important for their livelihood. Although their revenues were not considered significant, they found no serious reasons to switch to another activity.

In the framework of this study, adult loggers involved in the mangrove harvesting considered commercial exploitation of mangroves as a mean of poverty alleviation because of its high economic return. However, this does not apply to younger loggers who were willing to abandon logging because of difficulties (drowning risk, removal of logging sites, transport effort) associated to this activity. The average monthly income is given in the results section. If based on the so-termed US$1/day, loggers were not poor. However, consumers established near local markets (*e.g*. Youpwe market) were unable to pay for mangrove wood since their purchasing power was low (pers. obs.). Therefore, they were often involved in logging to meet domestic fuel and construction needs. This contributes to a relative progression of the mangrove loggers around the Wouri estuary. The same report is true for many coastal communities worldwide which are currently characterized by chronic poverty [11,13,15,33,35]. Din and Blasco [44] reported that harvesting has become more efficient because of the introduction of slicers and large dugouts propelled by engines. Both uses of chain saws and engine-powered canoes, as indicated by several loggers, should likely increased daily production and size of marketed woods and therefore loggers' revenues. As in many areas worldwide, harvesters were willing to venture widely in search of particular trees that have high local market value [9,13,19,45,46]. We found that *Rhizophora *spp. was more highly selected for this purpose.

Interestingly, our results indicate that the average monthly incomes of loggers were quite similar between age classes. This could be due to the fact that a wide range of harvesters, except older loggers who were less active, currently used both chain saws and slicers during logging activities. Furthermore, traditional boats usually have the same capacity that allows loggers to carry the same quantities of marketable wood (Figure 2A). However, small-scale mangrove cutting by older loggers is closely related to their common use of carpenter saws and machetes. The latter was different in a Venezuelan case where older mangrove users were found to be more experienced mangrove wood harvesters than younger ones [40].

We did not consider the impact of harvest frequency on loggers' monthly income since the wood quantity cut down was mainly influenced by the nature of logging materials (chain saw use accelerates mangrove loss more than slicers or machetes) (Table 4). This explains the fact that most of marketed wood diameter range from 40 cm to 60 cm. The wide range of sizes and species selectivity in cutting is closely related to the current increasing demand for commercial fuel-wood and charcoal in Douala city. Likewise, loggers' income was not significantly different between age classes (see results). This could also suggest that the wood prices in all markets investigated so far are not different. We did not made proper trend analyses of this hypothesis because of discrepancies among the form of marketed woods (e.g woods are sold in form of cubic meter or heap).

Taking into account the survey period and respondents' experience in logging activities, our results show that 87.5% of harvesters have started marketing wood during the 1990s. The high number of loggers with less than 10 years of experience in logging can be easily explained by the fact that towards the end of last century, several African countries, including Cameroon, were struck by serious socio-economic crises, which resulted in high levels of unemployment and widespread poverty [20]. Therefore, commercial exploitation of mangrove products became an important income supplement for coastal communities that are closest to the Wouri estuary.

Considering the fact that other anthropogenic pressures (especially urbanization) provoked an irreversible degradation, the luxuriant mangrove forest of the Cameroon Estuary with its current estimated surface area of about 1,000 km^2 ^has < 100 years left before being entirely converted into plantings without *Rhizophora *trees [11,20,41]. This corresponds well with Duke's [4] prediction of a world without mangroves.

### Subsistence uses

The use of considerable amounts of fuelwood, particularly from *Rhizophora*, as *banda *for smoking fish, rather than just for cooking or charcoal as reported in many other countries among which Kenya, India and Malaysia [3,9,47], is unique for the Cameroon case. This important activity (fish smoking) around the Douala-Edea reserve was regarded as a local fish preservation technique.

The use of *Laguncularia racemosa *as a construction material, particularly for fences, has also been recorded in the region of the Mexican Pacific coast by Hernández Cornejo et al. [48], who reported additional medicinal use of *Avicennia germinans *(e.g tea made from the leaves of *Avicennia germinans *was used for the treatment of gastric diseases) that was not found in the present study. Although the use of *Nypa fruticans *as thatching material for house walls and roofs has been reported at Mbiako, Yoyo I and Yoyo II, others non-wood mangrove uses (food, fodder, alcohol, sugar, medicine and honey) found worldwide [3,9,15,31,48] were not mentioned here.

Because they are geographically isolated and surrounded only by mangrove forests, the villagers reported that they did not know about alternatives to mangrove wood. Therefore, high household demands for firewood and housing have greatly affected plant populations. Because villagers were not also in good position to be selective, they would harvest what was most readily available to them [18]. This explains why mangrove ecosystem was of prime importance to most of the respondents (87%) despite the fact that it's declining.

## Conclusion

Around the Wouri estuary and the Douala-Edea reserve, local communities often depend on mangroves for subsistence and for commercial needs. Big logs with diameter greater than 40 cm were commonly sold by loggers at the Douala wood markets. However, loggers who were also involved in informal job practices often delivered logs to various resellers. Besides mangrove wood selling, *Rhizophora racemosa *and *R*. *harrisonii *were commonly used for fuelwood by villagers. The socio-economic value of mangroves in our study area is real and sufficiently important to merit greater interest from different stakeholders. Therefore, small-scale programmes of mangrove reforestation and afforestation should be recorded at the local levels especially where mangroves are irreversibly degraded. Furthermore, mangrove logging should be regulated through law enforcement, less use of chain saws, awareness and alternative jobs proposal.

## Competing interests

The authors declare that they have no competing interests.

## Authors' contributions

Author ANA made substantial contributions in drafting the manuscript, performed statistical analyses and made interpretation of data and finalized the manuscript with ND and FDG.

Author ND conducted field surveys and interviews with the loggers while SNL performed the interviews with the villagers. Both authors participated in the critical revision of the manuscript.

Author NK supervised the research works of SNL and revised the manuscript.

Author FDG participated in the design of the study and its coordination, and supervised SNL and ANA. He drafted parts of the manuscript and finalized the paper with ND and ANA.

All authors read and approved the final manuscript.
